# Late-Onset Esophageal Perforation After Salvage Anterior Cervical Spine Surgery in a Patient With Systemic Sclerosis

**DOI:** 10.7759/cureus.37831

**Published:** 2023-04-19

**Authors:** Kazuhiro Inomata, Eiji Takasawa, Tokue Mieda, Yoichi Iizuka, Hirotaka Chikuda

**Affiliations:** 1 Department of Orthopedic Surgery, Gunma University, Maebashi, JPN

**Keywords:** late-onset esophagus perforation, asymptomatic, anterior plate, salvage surgery, anterior cervical spine surgery, sclerotic skin, organ fibrosis, systemic sclerosis

## Abstract

Systemic sclerosis (SSc) is a systemic, immune-mediated disease characterized by abnormal cutaneous and organ-based fibrosis that results in progressive organ dysfunction including the esophagus. We herein report our experience of a patient with SSc who underwent salvage anterior cervical spine surgery that led to late-onset esophageal perforation.

A 57-year-old female had progressive cervical kyphosis after laminoplasty for cervical spondylotic myelopathy. We performed anterior cervical discectomy and fusion using a stand-alone cage. Despite prolonged use of a neck collar, migration of the anterior cage was noted three months after surgery. Rapid progression of kyphotic deformity prompted us to perform revision surgery of circumferential cervical correction. However, conventional posterior surgery was precluded due to the extremely poor condition of her neck, including severely sclerotic skin and atrophic muscles. To address this, she underwent a posterior fusion with the closed technique and C4-C5 corpectomy and bone grafting using a low-profile anterior plate. One year after the surgery, CT and routine upper gastrointestinal endoscopy (UGE) showed no evidence of esophagus injury. She remained asymptomatic thereafter. Over three years after her last surgery, however, follow-up CT incidentally revealed an abnormal air leak around the anterior plate. UGE demonstrated large esophagus perforation with the exposed metal plate. As she had already been on parenteral nutrition due to the disease progression of SSc, we decided not to remove the implant.

Potential esophagus perforation after anterior cervical spine surgery, even years later, should be considered regardless of the patient’s symptoms, such as chest pain and dysphagia. Spine surgeons need to be cognizant of the fragility of the esophagus, especially in patients with SSc. For patients with SSc, posterior reconstruction alone is recommended as a relatively safe option, even with a suboptimal skin condition.

## Introduction

Systemic sclerosis (SSc) is a systemic, immune-mediated disease characterized by abnormal cutaneous and organ-based fibrosis that results in progressive organ dysfunction including the esophagus [1,2]. We herein report our experience of a patient with SSc who underwent salvage anterior cervical spine surgery that led to late-onset esophageal perforation.

## Case presentation

A 57-year-old woman had diffuse cutaneous SSc and was taking prednisolone for its treatment. She was admitted for neurosurgery at our hospital due to progressive numbness and gait disturbance. MRI showed multiple-segment cord compression induced by a calcified tumor in the cervical spine. The patient underwent C3-C5 recapping laminoplasty with titanium mini-plates and screws to resect calcified lesions that were pathologically diagnosed as calcified flavum and a facet capsule. Her postoperative recovery was complicated by the slow healing of the surgical wound, which required over one year to be closed.

**Figure 1:**
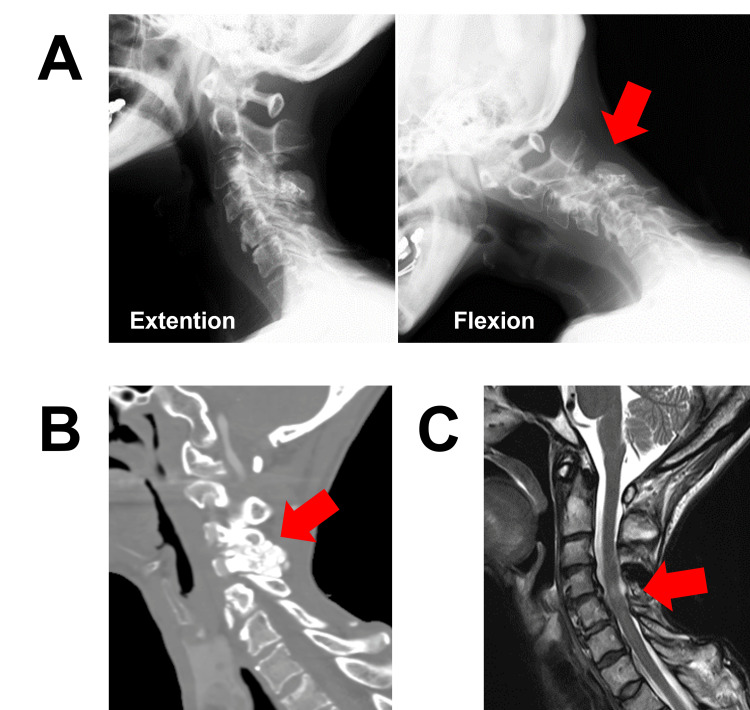
Preoperative images A) X-ray showed C4 anterior slip with dynamic instability. B) CT showed tumoral calcinosis of the connective tissues of the spine (ligamentum flavum and facet capsule). C) MRI showed intramedullary signal change at the C4/5 level.

Two years after her initial surgery, the patient was referred for orthopedic surgery at our hospital because of the resurgence of myelopathic symptoms, including numbness in her extremities, muscle weakness, and gait disturbance. Flexion and extension radiograph X-ray and CT showed C4 anterior slip with dynamic instability (Figure 1A-B). MRI also demonstrated intramedullary signal change at the C4/5 level (Figure 1C).

**Figure 2:**
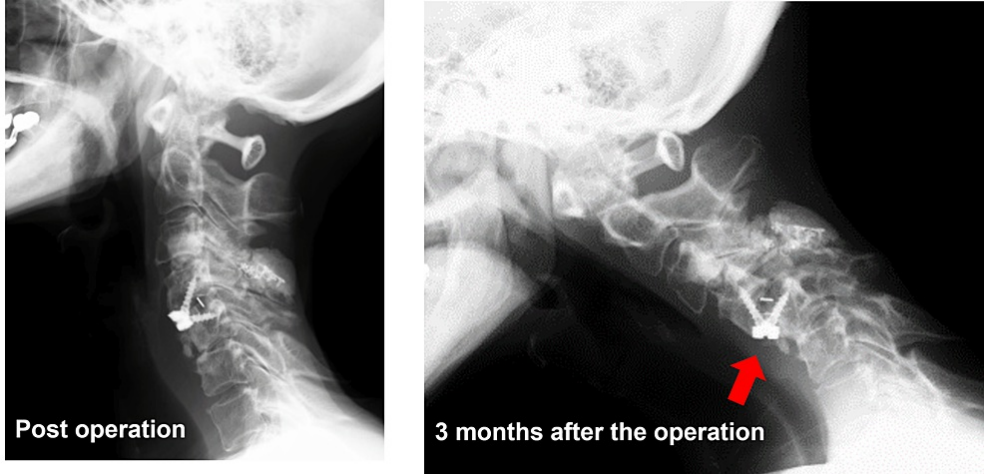
Images of anterior cervical decompression and fusion with the stand-alone cage Anterior cervical discectomy and fusion at the C4/5 level were performed using a stand-alone cage. Migration of the anterior cage was noted three months after surgery.

The extremely poor condition of her neck, including severely sclerotic skin and atrophic muscles, precluded conventional posterior surgery. To address this, anterior cervical discectomy and fusion were performed using a stand-alone cage. Despite prolonged use of a neck collar, migration of the anterior cage was noted three months after surgery (Figure 2).

**Figure 3:**
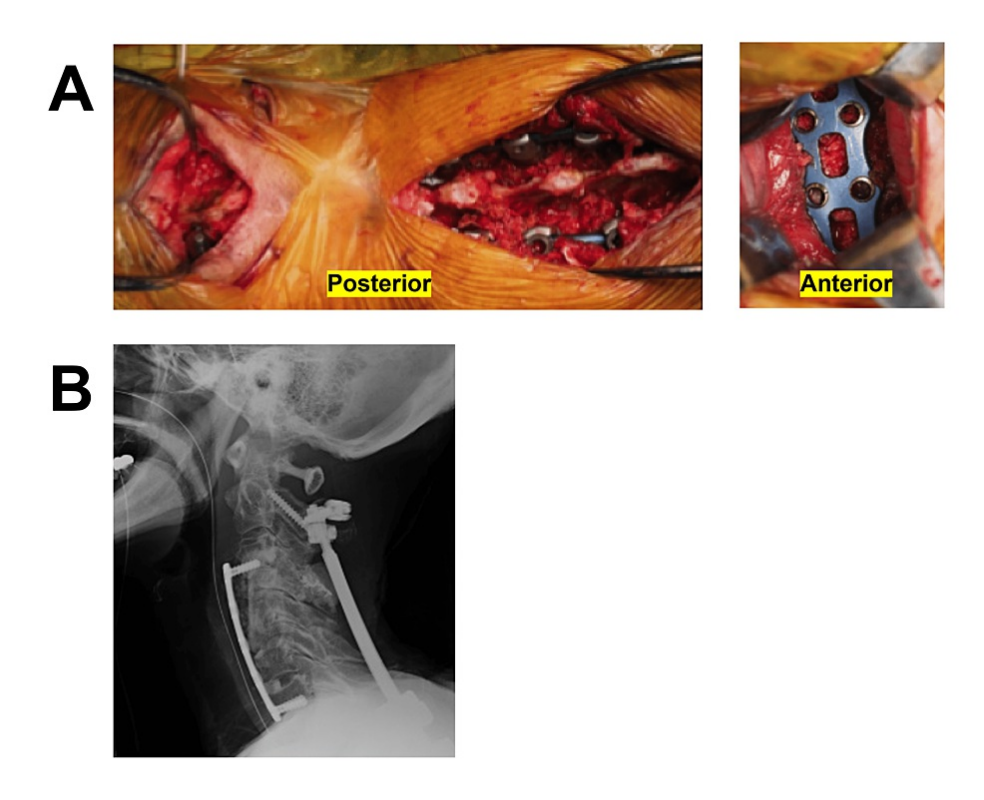
Images of revision surgery A) For posterior correction and fusion, lamina or pedicle screws were inserted into the vertebrae above and below the skin lesion at the C3-C6 levels (i.e., C2, T2, T3, and T4 vertebrae). Titanium alloy rods were subcutaneously applied in a closed technique. C4-C5 corpectomy and fibula bone grafting were performed with anterior plating at the C3-C7 levels. B) Postoperative radiograph.

Rapid progression of kyphotic deformity prompted us to perform revision surgery of circumferential cervical correction. For posterior correction and fusion, lamina or pedicle screws were inserted into the vertebrae above and below the skin lesion at the C3-C6 levels. Dual-diameter titanium-alloy rods (φ4.0-5.0 mm) were subcutaneously applied in a closed technique. After posterior surgery, the patient was repositioned, and anterior surgery was performed. We performed C4-C5 corpectomy and fibular bone grafting using a low-profile anterior plate (Figure 3A-B).

**Figure 4:**
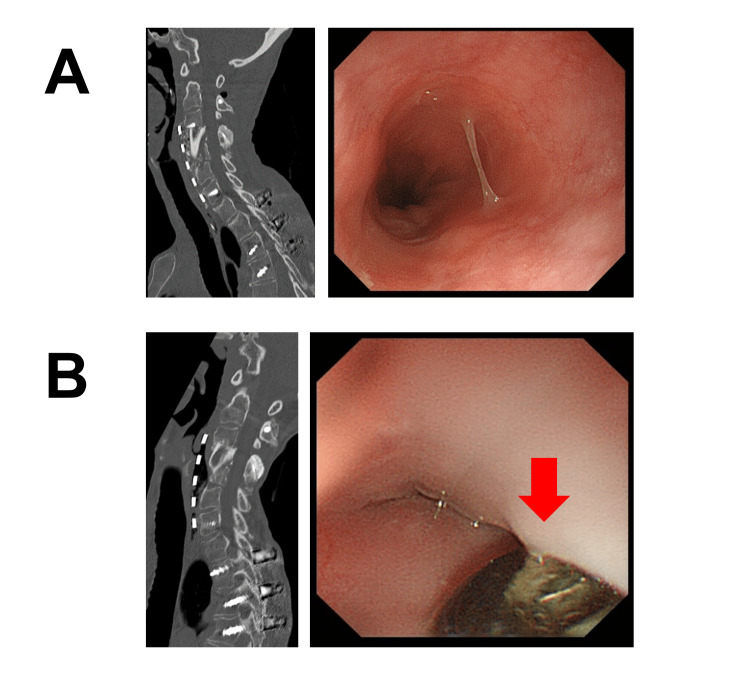
Follow-up CT and UGE Images at one year (A) and over three years (B) after surgery.

Meticulous attention was paid to avoid damaging her esophagus throughout the procedure. We were unable to cover the plate completely with adjacent soft tissue because of the poor skin condition. Her neurological symptoms improved postoperatively. One year after the surgery, CT and routine upper gastrointestinal endoscopy (UGE) showed the proper position of the anterior plate and no evidence of esophagus injury (Figure 4A). She remained asymptomatic thereafter.

Over three years after her last surgery, however, follow-up CT incidentally revealed an abnormal air leak around the anterior plate. Bony fusion was achieved at the C3-C7 levels, and there were no implant failures, including screw loosening or backout. UGE demonstrated large esophagus perforation with the exposed metal plate (Figure 4B). As she had already been receiving parenteral nutrition due to the disease progression of SSc for three years, we decided not to remove the implant. At the last follow-up, the patient remained ambulatory with no sign of implant infection or mediastinitis.

## Discussion

Esophageal perforation in anterior cervical spine surgery is rare, with a prevalence of 0%-1.6%; however, it can be fatal in up to 16% of cases [3]. Delayed esophagus perforation is related to chronic compression, dislocation, or migration of the grafts or internal fixation materials, while an early lesion is caused by direct iatrogenic injuries, such as retraction or improper placement of plates, screws, or bone grafts [3,4]. Esophagus perforation can occur over 20 years after surgery, suggesting that careful long-term follow-up is required [5,6].

Involvement of the gastrointestinal tract (GIT) in SSc is extremely frequent; it is a leading cause of morbidity and the most common cause of mortality in this disease. Esophageal abnormalities occur in up to 90% of cases. The pathogenesis of GIT involvement is thought to include early vascular damage to the vasa nervorum of the nerves innervating the GIT. This leads to neurological dysfunction. With damage to innervation, the smooth muscle atrophies and is eventually replaced by fibrotic tissue. Furthermore, ineffective esophageal motility contributes to mucosal damage secondary to refractory acid reflux [7]. Thus, the esophageal wall in patients with SSc is thin, fragile, and chronically inflamed, and sustained stimulation with metal implants can cause esophagus damage. Esophagus perforation can cause chest pain and dysphagia; however, up to 30% of esophageal disorders and perforations in patients with SSc are asymptomatic [3,5]. As in the present case, parenteral nutrition may mask such clinical signs and result in a delayed diagnosis. Since the patient was unable to receive oral intake and was instead receiving intravenous nutrition, she was able to be observed without treatment for perforation with no fatal complications. However, we should exercise caution, as esophageal perforation can cause mediastinitis, which can be fatal.

## Conclusions

Potential esophagus perforation after anterior cervical spine surgery, even years later, should be considered regardless of a patient’s symptoms. Spine surgeons need to be cognizant of the fragility of the esophagus when performing anterior cervical surgery, especially in patients with SSc. For patients with SSc, posterior reconstruction alone is recommended as a relatively safe option, even with a suboptimal skin condition.
